# Serum Vitamin D Concentrations Are Associated With Depressive Symptoms in Men: The Sixth Korea National Health and Nutrition Examination Survey 2014

**DOI:** 10.3389/fpsyt.2020.00756

**Published:** 2020-07-30

**Authors:** Sang Jin Rhee, Hyunju Lee, Yong Min Ahn

**Affiliations:** ^1^ Department of Neuropsychiatry, Seoul National University Hospital, Seoul, South Korea; ^2^ Department of Psychiatry, Seoul National University College of Medicine, Seoul, South Korea; ^3^ Institute of Human Behavioral Medicine, Seoul National University Medical Research Center, Seoul, South Korea

**Keywords:** depressive symptoms, Patient Health Questionnaire 9, vitamin D, negative binomial distribution, men

## Abstract

**Objective:**

There is increasing evidence of an inverse association between serum vitamin D concentrations and depression, but whether there are sex-specific differences remains controversial. Thus, the aim of this study was to investigate the association between serum vitamin D concentrations and specific domains of depressive symptoms by each sex in the Korean general population.

**Methods:**

The study sample comprised 820 men and 916 women, aged from 19 to 76, who participated in the Korea National Health and Nutrition Examination Survey 2014. Participants completed health interviews and health examinations providing data of serum 25-hydroxyvitamin [25(OH)D] concentrations, the Patient Health Questionnaire-9 (PHQ-9), and certain covariates. Associations were analyzed using negative binomial regression.

**Results:**

After adjusting for various covariates, the association between log-transformed serum 25(OH)D concentrations and total PHQ-9 scores was statistically significant {incidence rate ratio [IRR] = 0.74 [95% confidence interval (CI) = 0.59–0.93]} only in men. Additionally, the association between log-transformed serum 25(OH)D concentrations and the PHQ-9 cognitive/affective subscore was statistically significant [IRR = 0.56 (95% CI = 0.40–0.80)] only in men. There was no association for the somatic subscore.

**Conclusions:**

Serum vitamin D levels were inversely associated with cognitive/affective depressive symptoms only in men.

## Introduction

Interest in the role of micronutrients in health and diseases is increasing. Vitamin D, especially, is not only known for its involvement in calcium/phosphate homeostasis and bone health, but also in various diseases, such as diabetes, dyslipidemia, and cancer (1, 2). Through exposure to sunlight, 7-dehydrocholesterol is converted to cholecalciferol (vitamin D3), which is the major source of vitamin D in the human body (3). 25-hydroxyvitamin D [25(OH)D] is the product of 25-hydroxylation of vitamin D3, and is the major storage of vitamin D and the most reliable marker of vitamin D status (3, 4). Interestingly, metabolites of 25(OH)D can cross the blood-brain barrier and bind to vitamin D receptors that are widely distributed in the central nervous system (5), which emphasizes its association with mental health, including depression.

A vast number of studies on the association between serum 25(OH)D concentration and depressive symptoms have been conducted, and although there have been some negative results, numerous number of studies have shown positive results (6, 7). However, whether there are sex differences regarding this association is still controversial. Population-based studies in Norway and Italy have shown that the association is stronger in women (8, 9), while a study in Finland reported that the association is stronger in men (10), and studies in Australia and the Republic of Korea found that the association is only significant in men (11, 12). Further studies have investigated with more specificity and have shown that the association of serum 25(OH)D and depressive symptoms are more robust for pregnant women (13). Additionally, the few studies in patients with depressive episodes that have investigated the relationship between serum 25(OH)D and certain domains of depressive symptoms showed differing results (14, 15). Analyzing the association with regard to specific domains of depressive symptoms in the general population may give us more information on the underlying mechanisms.

Thus, this study was conducted to analyze the association between serum 25(OH)D concentrations and depressive symptoms in the Korean general population. Because there have been earlier reports on sex differences in serum 25(OH)D concentrations (16–18), we analyzed men and women who took part in the Korea National Health and Nutrition Examination Survey (KNHANES) VI-2 (2014) separately. We hypothesized that serum 25(OH)D concentrations and depressive symptoms are inversely associated, and that men and women show different associations with cognitive/affective and somatic domains of depressive symptoms.

## Materials and Methods

### Study Sample Population

The KNHANES is a national representative survey that is being conducted annually by the Division of Health and Nutrition Survey, Korea Centers for Disease Control and Prevention (KCDC). It is a representative sample of noninstitutionalized civilians in the Republic of Korea and is designed to assess health and nutritional status and estimate the prevalence of chronic diseases (19). The KNHANES uses a complex, multi-stage probability sample design (19). Using data from all households collected during the Population and Health Census of Korea in 2010, 192 primary sampling units (PSUs) were selected based on administrative districts and housing types. A systemic sampling system with intra-stratification of age, sex, residential area, and the educational background of the head of the household was applied to extract 20 households from each PSU. Finally, within each household, individuals aged 1 year and over were selected to participate.

We used data from the health interviews and health examinations from the KNHANES VI-2 (2014), which was conducted from January to December 2014. Self-administered and structured questionnaires were used to collect information about demographic and clinical characteristics and physical examinations were done *via* household interviews and at mobile examination centers. The total number of participants in the KNHANES VI-2 was 7,550. Patient Health Questionnaire-9 (PHQ-9) was administered to those who participated in the health interview and were at least 19-years-old. Serum 25(OH)D concentrations was targeted to analyze a subsample of 2,400 participants for those who were at least 10-years-old. Those with full serum 25(OH)D data and a completed Patient Health Questionnaire-9 (PHQ-9) were eligible, which yielded a total of 1,825 participants (859 men, 966 women), with an age range from 19 to 76. We excluded women who were pregnant (n=8) or lactating (n=9), and those who were currently on treatment for depression (7 men, 16 women) or had missing data for covariates (32 men, 17 women). The final study population consisted of 1,736 (820 men, 916 women) participants.

The survey procedures were approved by the Korea Centers for Disease Control and Prevention Institutional Review Board (No. 2013-12EXP-03-5C) and the methods were carried out in accordance with the approved guidelines. Written informed consent was obtained from all survey participants. Additional ethical approval for the study design was waived due to the publicity/anonymity of the data.

### Assessment of Serum 25(OH)D Concentrations

After at least 8 h of fasting, blood samples were collected to assess levels of biochemical markers. Serum concentrations of 25(OH)D were measured from venous samples that had been collected in a serum separating tube and centrifuged at 3,000 rpm for 15 min. The concentration was measured with a 25-Hydroxyvitamin D 125I RIA Kit (DiaSorin, Stillwater, MN, USA) using a 1470 WIZARD Gamma Counter (PerkinElmer, Turku, Finland). The levels were analyzed by the institute (NeoDin Medical Institute, Seoul, Republic of Korea) that participated in the international Vitamin D External Quality Assessment Scheme (DEQAS), and the DEQAS proficiency testing showed a standard deviation index of less than ± 2.0 in 2014 (17). The NIST (National Institute of Standards and Technology) developed the standardized reference material (SRM927a) that was used for the traceability tests; the bias was under 10% except for level 2 (low concentration; 18.91 ng/ml, bias: 14.91%) (17). The coefficient of variation for the control material was set at <6.6% for low and <5.8% for high levels and was satisfied (17).

### Assessment of Depressive Symptoms

The severity of depressive symptoms was assessed with the PHQ-9. The PHQ-9 is a 9-item self-report questionnaire that scores each question from 0 to 3 points based on the frequency of each symptom (total score = 0–27 points), and is generally used to assess the severity of depressive symptoms (20). The suggested cutoff for mild, moderate, moderately severe, and severe depressive symptoms are 5, 10, 15, and 20 points, respectively (20). Although it is not a structured diagnostic interview, this brief self-report questionnaire is commonly used for screening depression, and the cutoff of 10 points is known to have appropriate sensitivity and specificity for major depressive disorder (20, 21). The Korean version of the PHQ-9 was used in the survey. Previous studies have reported significant internal consistency (α = 0.81–0.86) and test-retest reliability (coefficient = 0.79–0.89) (22, 23). The total score of the PHQ-9 was initially analyzed as the dependent variable in regression models, since the number of participants with at least 10 points of the PHQ-9 was insufficient to analyze PHQ-9 as a dichotomous dependent variable (based on the cutoff of 10 points) considering the need to control for numerous covariates. A two-dimensional structure was applied based on previous studies, the cognitive/affective and the somatic domain, and total subscores of these domains were further analyzed (24, 25). The items “loss of interest”, “depressive mood”, “feeling of worthlessness”, “trouble concentrating”, and “suicidal thoughts” were comprised in the cognitive/affective domain, while “sleep problems”, “fatigue or loss of energy”, “change in appetite”, and “psychomotor retardation or agitation” were comprised in the somatic domain.

### Other Covariates

Questionnaires were used to collect sociodemographic information, lifestyle behaviors, and health factors. These included sex, age, region, income, education, marriage experience, occupation, average sleep time, alcohol use, smoking behavior, and aerobic exercise. The analysis was stratified by sex. Age was treated as a continuous variable while region (rural or urban), income (less than the 1^st^ quartile of housing income or at least the 1^st^ quartile of housing income), education (not a college graduate or at least a college graduate), marriage experience (currently/previously married or never married), and occupation (employment or unemployment) were treated as dichotomous variables. Average sleep time was subclassified into three groups (5 h or less, 6–8 h, 9 h or more). Alcohol use (yes or no), smoking behavior (current smoker or current non-smoker), and aerobic exercise (yes or no) were also defined as dichotomous variables. Alcohol use was defined as drinking at least one cup of alcoholic beverages per month for 1 year, and a current smoker was defined as someone who was currently smoking and had smoked at least 100 cigarettes throughout their lifetime. Aerobic exercise was defined as physical activity of moderate intensity for at least 2 ½ h per week or physical activity of high intensity for at least 1 ¼ h per week (high-intensity physical activity was given doubled weight compared to moderate-intensity physical activity for those who engaged in both intensities).

Examination season was dichotomously classified as winter (December–February) or non-winter. Hypertension was defined by systolic blood pressure ≥ 140 mmHg, diastolic blood pressure ≥ 90 mmHg, or use of antihypertensive medication. Diabetes was defined by fasting glucose ≥ 126 mg/dl, a diagnosis by a doctor, or use of antidiabetic medication including insulin injections. Hypercholesteremia was defined by fasting total cholesterol ≥ 240 mg/dl or antilipidemic medication (26). Obesity was defined by body mass index (BMI) ≥ 25 kg/m^2^ (27), which was calculated by dividing measured body weight (kilograms) by body height (meters) squared. Anemia was defined by hemogloblin < 12 g/dl for women and < 13 g/dl for men. The definitions for these conditions were based on the guideline of the KNHANES. A history of cancer was based on the participant’s self-report. All of these variables were treated as dichotomous variables. Finally, serum laboratory findings that were used as continuous covariates were AST (aspartate aminotransferase) levels, free T4 levels, and creatinine and, due to the skewed distribution, a logarithmic transformation was applied.

### Statistical Analysis

As the KNHANES has a complex sample design, sampling procedures and weights were adjusted for the following analyses. Demographic and clinical characteristics of the study population were analyzed and compared between men and women using chi-square tests for categorical variables, t-tests for continuous variables except PHQ-9 scores, and Kruskall Wallis tests for PHQ-9 scores. Initially the log-transformed levels of serum 25 (OH)D between those with at least moderate depressive symptoms and those with absent/mild depressive symptoms were compared with t-tests. As the distribution of PHQ-9 scores was positively skewed and contained, similar to count data, an excess of zeros (33.7% in the final population), we used negative binomial regression to analyze the association between the log-transformed serum 25(OH)D concentrations as the independent variable and the continuous total scores and subscores of the PHQ-9 as the dependent variable. Before stratification, the statistical significance of the interaction between log-transformed serum 25(OH)D and sex for PHQ-9 data was assessed. The multivariate analysis was then stratified by sex, and the covariates previously mentioned were all controlled for. Model fitness was calculated by dispersion, and weights were standardized to the sample size of each sex by dividing each person’s weight by the mean weight of their sex.

All statistical tests were two-sided, and p <.05 was considered to be statistically significant. Statistical analyses were mainly performed with SPSS Version 21 (IBM Corporation, Armonk, NY, USA). STATA Version 10 (Stata Corp, College Station, TX, USA) was used for the negative binomial regression, and R Version 3.5.0 was used for the Kruskall Wallis test.

## Results

### Demographic and Clinical Characteristics for Each Sex

Demographic and clinical characteristics were compared between male and female study participants (**Table 1**). Alcohol use, smoking, hypertension, diabetes, and obesity were significantly more common in men, and low income, less education, unemployment, and anemia were significantly more common in women. The serum laboratory tests showed that AST, free T4, creatinine, and 25(OH)D levels were all higher in men. PHQ-9 scores were higher in women. Ninety-six participants’ (5.2% in the final population regarding the complex sample design) PHQ-9 scores were at least 10 points. The prevalence was 7.7% in women, and 3.0% in men.

**Table 1:** Demographic and clinical characteristics of the study subjects by sex.

	Men^a^ (n=820, Size=12,146,172)	Women^a^ (n=916, Size=10,378,188)	Statistics^a^ ^,^ ^b^	df	P-value^a^ ^,^ ^c^
Age, mean (95% CI), years	42.20 (41.21–43.19)	42.63 (41.68-43.58)	t=0.68	167	0.50
Rural region, *n* (%)	143 (16.0%)	139 (14.2%)	F=1.00	1	0.32
Less than the 1st quartile of housing income, *n* (%)	78 (7.7%)	120 (12.0%)	F=9.34	1	0.003**
At least a college graduate, *n* (%)	329 (40.4%)	297 (32.5%)	F=9.80	1	0.002**
Never married, *n* (%)	216 (31.4%)	207 (25.8%)	F=5.39	1	0.21
Unemployment, *n* (%)	179 (21.6%)	419 (45.7%)	F=94.86	1	<0.001***
Average sleep time			F=2.79	1.99	0.06
≤ 5 h, *n* (%)	101 (11.8%)	135 (14.5%)			
6–8 h, *n* (%)	670 (82.6%)	710 (77.6%)			
≥ 9 h, *n* (%)	49 (5.6%)	71 (8.0%)			
Alcohol use, *n* (%)	612 (75.6%)	421 (47.0%)	F=104.52	1	<0.001***
Current smoker, *n* (%)	355 (44.3%)	50 (5.9%)	F=239.88	1	<0.001***
No aerobic exercise, *n* (%)	322 (37.4%)	399 (41.4%)	F=2.69	1	0.10
Winter examination season, *n* (%)	176 (20.9%)	202 (22.8%)	F=1.15	1	0.29
Hypertension, *n* (%)	224 (22.9%)	160 (15.8%)	F=11.54	1	0.001**
Diabetes, *n* (%)	85 (8.2%)	55 (5.2%)	F=6.84	1	0.01*
Hypercholesteremia, *n* (%)	102 (11.2%)	118 (11.1%)	F=0.01	1	0.91
Obesity, *n* (%)	323 (37.8%)	243 (25.2%)	F=24.96	1	<0.001***
Anemia, *n* (%)	25 (2.3%)	74 (7.6%)	F=17.03	1	<0.001***
History of cancer, *n* (%)	25 (2.4%)	42 (4.9%)	F=6.51	1	0.12
AST^d^, mean (95% CI), IU/L	22.05 (21.46–22.66)	18.81 (18.43–19.19)	t=−10.02	167	<0.001***
free T4^d^, mean (95% CI), ng/dl	1.29 (1.27–1.30)	1.20 (1.19–1.21)	t=−8.23	167	<0.001***
Creatinine^d^, mean (95% CI), mg/dl	0.94 (0.93–0.95)	0.70 (0.69–0.70)	t=−41.96	167	<0.001***
25(OH)D^d^, mean (95% CI), ng/ml	15.41 (14.83–16.02)	13.81 (13.32–14.31)	t=−5.61	167	<0.001***
PHQ-9 total score ≥ 10, *n* (%)	27 (3.0%)	69 (7.7%)	F=14.93	1	<0.001***
PHQ-9 total score, median (interquartile range)	1 (0–3)	2 (0–5)	t=6.59	166	<0.001***
PHQ-9 cognitive/affective subscore, median (interquartile range)	0 (0–1)	0 (0–2)	t=3.79	166	<0.001***
PHQ-9 somatic subscore, median (interquartile range)	1 (0–2)	1 (0–3)	t=4.82	166	<0.001***

CI, confidence interval; AST, aspartate aminotransferase, 25(OH)D, 25-hydroxyvitamin D; PHQ-9, Patient Health Questionnaire-9.

a Statistics adjusted for complex sample design.

b Categorical variables based on adjusted F by Pearson’s chi-square tests, continuous variables except PHQ-9 scores based on t tests, PHQ-9 scores based on design-based Kruskal Wallis tests.

c P-values are significant at α =0.05 (*), 0.01 (**), and 0.001 (***).

d Due to skewed distribution, logarithmic transformation was performed for the statistics, CIs were back-transformed.

### Association Between Serum 25(OH)D Concentrations and Depressive Symptoms

In men, the log-transformed serum 25(OH)D concentration was lower in those with at least moderate depressive symptoms than those with absent/mild depressive symptoms (3.57 ± 0.10 vs 4.01 ± 0.04, t = −4.18, df = 167, P < 0.001). However, in women there was no significant difference (3.81 ± 0.13 vs 3.79 ± 0.04, t = 0.17, df = 167, P = 0.86).

The interaction term between log-transformed serum 25(OH)D concentrations and sex was significantly associated with total PHQ-9 scores (P = 0.039), and the analysis was thus stratified by sex. Adjustments were applied for age, region, income, education, marriage experience, occupation, average sleep time, alcohol use, smoking behavior, aerobic exercise, examination season, hypertension, diabetes, hypercholesteremia, obesity, anemia, history of cancer, AST, free T4, and creatinine. In the multivariate analysis of male participants, the association between log-transformed serum 25(OH)D concentrations and total PHQ-9 scores was statistically significant {incidence rate ratio [IRR] = 0.74 [95% confidence interval (CI) = 0.59–0.93], df = 167, P = 0.011}. Additionally, in the subscore analysis, the association between log-transformed serum 25(OH)D concentrations and the PHQ-9 cognitive/affective subscore was statistically significant [IRR = 0.56 (95% CI = 0.40–0.80), df = 167, P = 0.002]; however, the association with the PHQ-9 somatic subscore was not significant [IRR = 0.85 (95% CI = 0.68–1.06), df = 167, P = 0.15]. In the multivariate analysis of female participants, log-transformed serum 25(OH)D concentrations were not significantly associated with the total scores or the subscores of the PHQ-9 (**Table 2**).

**Table 2:** The association between serum 25-hydroxyvitamin D concentrations and depressive symptoms by negative binomial regression.

	Men					Women				
	(n=820, Size=12,146,172)					(n=916, Size=10,378,188)				
	Incidence Rate Ratio (95% CI)	Design df	P-value^a^	Alpha	Dispersion^b^	Incidence Rate Ratio (95% CI)	Design df	P-value^a^	Alpha	Dispersion^b^
Total score analysis^c^										
PHQ-9 total score	0.74 (0.59–0.93)	167	0.011*	1.22	0.98	1.03 (0.80–1.33)	167	0.83	1.06	0.98
Subscore analysis^c^										
PHQ-9 cognitive/affective subscore	0.56 (0.40–0.80)	167	0.002**	2.17	0.99	1.01 (0.71–1.43)	167	0.97	2.03	0.96
PHQ-9 somatic subscore	0.85 (0.68–1.06)	167	0.15	0.81	1.02	1.03 (0.82–1.28)	167	0.80	0.70	0.99

CI, confidence interval; PHQ-9, Patient Health Questionnaire-9.

a P-values are significant at α =0.05 (*), 0.01 (**).

b Dispersion based on Pearson.

c Adjusted for age, region, income, education, marriage experience, occupation, average sleep time, alcohol use, smoking behavior, aerobic exercise, examination season, hypertension, diabetes, hypercholesteremia, obesity, anemia, history of cancer, AST, free T4, and creatinine.

## Discussion

After controlling for various confounding factors, in our study sample of 820 men and 916 women who participated in the KNHANES VI-2 (2014), lower serum vitamin D concentrations were significantly associated with higher PHQ-9 scores only in men. As for specific domains, this association was significant for the cognitive/affective domain of the PHQ-9. No association was observed in women.

The prevalence for those with at least moderate depressive symptoms (PHQ-9 ≥10) in the final population was 5.2%. The prevalence was lower than the population studies which used depressive symptoms as an outcome (9, 10, 12) and similar to the study which used major depressive disorder as an outcome (10). Different age populations and different tools to evaluate relevant depressive symptoms/major depressive disorder would have influenced the discrepancy. Moreover, the study was based on a non-institutional population in the Republic of Korea who agreed to participate, and those who were on treatment with depression were excluded, which would represent a rather healthy group.

Importantly, the finding that an inverse association between serum vitamin D levels and depressive symptoms is confined to men replicates some earlier population-based studies (11, 12). However, other population-based studies found a significant association for both sexes, some with a stronger association in women (8, 9), others in men (10). Differences in study populations and designs can probably explain these discrepancies. Two of these studies were confined to participants over 65 years of age (9, 12), and one study only comprised 20-year-old participants (11). The remaining two studies had a broad age range, but only included participants who were at least 30 years old (8, 10). Our study age range was also broad, but our participants were younger than those of some of the previous studies, which could have influenced the associations we observed. However, the two studies confined to participants over 65 years also showed discrepancies in their results. Ethnicity and geological region could be alternative explanations as these studies were all from different countries. Interestingly, our study replicated the results of one earlier study in a Korean sample population that also found the same association only in men (12). Finally, differences in the statistical analyses that were used could have led to the observed discrepancies. Most studies focused on depressive symptoms/depression as a dichotomous variable assessed *via* logistic regression, which could lead to certain cutoff issues. Only one earlier study (from Australia) used negative binomial regression reflecting the distribution of depressive symptom scale scores; our study replicated the results of that study, showing that the association is confined to men (11).

Only few studies have assessed the relationship between vitamin D levels and specific depressive symptoms or domains. One earlier study analyzed 380 inpatients with depressive episodes and found that vitamin D deficiency was associated with cognitive/affective but not somatic/affective symptoms (15). Another study retrospectively assessed the data of 196 patients with a major depressive episode and reported that vitamin D insufficiency/deficiency was not associated with items related to anxiety, insomnia, and somatization, but with items related to depressive factors (depressive mood, anhedonia, suicidal ideation, and work/activities) (14). A recent study showed that hypovitaminosis D and cognitive inhibition impairment is associated in those with major depressive episodes (28). Our study, based on the general population, showed that there is a sex-specific, inverse association between serum vitamin D levels and cognitive/affective subscores of depressive symptoms. However, the previous studies did not stratify analyses by sex and only controlled sex as a covariate. Furthermore, they were based on a different study population, that is, patients with depressive episodes, which limits direct comparisons.

There are several potential explanations for the sex-specific association. First, in men, Vitamin D deficiency is known to be associated with lower testosterone (29), and these lower levels of testosterone are known to be associated with depression (30). Testosterone could thus be a mediator between the association of vitamin D deficiency and depression. However, in women, vitamin D deficiency is associated with high androgen level states like polycystic ovary syndrome, which is also known to have higher depression incidences (31). Additionally, another study finding a stronger association between vitamin D deficiency and depression in women has suggested a mediating role of estrogen (8). Second, metabolic/inflammation functions could be regulated in sex-specific ways. In a recent animal study, male mice were observed to have greater insulin resistance than female mice with 25(OH)D deficiency (32). It has also been shown CRP levels are higher only in male depressive subjects (33). Such a sex-specific difference in metabolic/inflammation functions could explain the observed sex-specific association. However, there is evidence suggesting that these metabolic risks are associated with somatic depressive symptoms (34). Third, the effect of chronic stress in depression could provide more information on the role of vitamin D, as it is known as a relevant risk factor of cognitive/affective depressive symptoms (35). Chronic stress induced dendritic atrophy in hippocampal pyramidal neurons (36), and affected the regulation of Interleukin 1 beta (IL-1ß) (37), only in male rats. Additionally, social isolation stress tests induced anhedonia-like behavior only in male rats, and reductions in synaptic proteins and spine density after social isolation were reversed by ketamine only in male rats (38). Vitamin D metabolites could have specific roles in these sex-specific changes in the central nervous system. Specifically, depression can stem from an imbalance between excitatory and inhibitory neurons, which is a result of increased Ca^2+^ in neuronal cells, and vitamin D deficiency is known to increase intracellular Ca^2+^ (39). Little is known about sex-specific differences in intracellular Ca^2+^ and its association with depression, except for findings from some gene studies. The *CACNA1C* gene, which encodes the alpha-1 subunit of the Ca_v_1.2 L-type voltage-dependent gated Ca^2+^ channel, is one of the most commonly reported genes in depression association studies (40, 41); increased activity of this channel results in increased neuronal Ca^2+^ levels (39). Interestingly, there is increasing evidence that sex should be considered in associations with depression, as polymorphisms of this gene are known to be sex-specific (42). Further investigations of sex-specific differences in intracellular Ca^2+^ and depression are thus needed for clarification of the underlying mechanisms.

The present study has several limitations. First, it has a cross-sectional design and causality therefore cannot be determined. There is a possibility that depressive symptoms are a cause of lower serum vitamin D concentrations. Depressive individuals are more likely to go out less and are thus less exposed to sunlight, which could lead to lower serum vitamin D concentrations. Second, there are other confounding factors that were not controlled for. For instance, dietary intake of vitamin D or sunlight exposure were not measured, and other psychiatric disorders including dementia and anxiety disorders were not evaluated. Additionally, there is evidence that PTH (parathyroid hormone) might also be a confounding factor in the associations assessed here (43). However, it is noteworthy that there is evidence of a negative association between PTH and depression (44). Third, the assessment of depressive symptoms was based on a self-reported scale and not on a comprehensive psychiatric evaluation. However, the PHQ-9 is based on the diagnostic criteria of major depressive disorder as described in the Diagnostic and Statistical Manual of Mental Disorders (DSM), and it has been shown to have significant internal consistency and test-retest reliability. Fourth, as the analysis was based on the Korean general population, generalizability to other ethnic groups and geographical areas is limited.

Despite these limitations, the present study also has a number of strengths. First, the data was based on a broad age range sample taken from the Korean general population, which represents an ethnically homogeneous group and a geographically homogeneous region; both of these factors are known to affect vitamin D concentrations (45). Second, the analysis was stratified by sex and assessed specific domains of depressive symptoms, extending our understanding of these associations. Third, we used negative binomial regression adjusting for complex survey designs to reflect the distribution of depressive symptom scores in the general population, and used continuous variables to yield sufficient statistical power and clear dose-response relationships.

In conclusion, our study shows that serum vitamin D levels are inversely associated with depressive symptoms, especially with the cognitive/affective domain, but only in men. This emphasizes the importance of screening for and diagnosing depression along with laboratory evaluations of vitamin D levels in men. Further research including longitudinal studies with multiethnic samples and evaluations of specific mechanisms should consider sex differences and specific depressive symptom domains.

## Data Availability Statement

The data analyzed in this study is subject to the following licenses/restrictions: This study analyzed the data from the KNHANES VI-2 (2014). The data is available to the public with the exception for data of examination season which requires further procedures to access. (http://knhanes.cdc.go.kr). Requests to access these datasets should be directed to http://knhanes.cdc.go.kr.

## Ethics Statement

The survey procedures were approved by the Korea Centers for Disease Control and Prevention Institutional Review Board (No. 2013-12EXP-03-5C) and the methods were carried out in accordance with the approved guidelines. Written informed consent was obtained from all survey participants. Additional ethical approval for the study design was waived due to the publicity/anonymity of the data.

## Author Contributions

Drafting of the manuscript: SR. Review and editing the manuscript: HL and YA. Analysis: SR. Study design: SR, HL, and YA. All authors contributed to the article and approved the submitted version. The authors are entirely responsible for the scientific content of this paper.

## Conflict of Interest

YA receives research support from or serves as a speaker for Janssen Korea Ltd., Lundbeck Korea Co., Ltd, and Korea Otsuka Pharmaceutical.

The remaining authors declare that the research was conducted in the absence of any commercial or financial relationships that could be construed as a potential conflict of interest.

## Abbreviations

25(OH)D, 25-hydroxyvitamin D; KNHANES, Korea National Health and Nutrition Examination Survey; PSU, primary sampling units; PHQ-9, Patient Health Questionnaire-9; DEQAS, Vitamin D External Quality Assessment Scheme; IRR, incidence rate ratio; CI, confidence interval
